# Supplementary material for: The BCG Moreau Vaccine Upregulates In Vitro the Expression of TLR4, B7-1, Dectin-1 and EP2 on Human Monocytes
Source: Vaccines (Basel). 2022 Dec 30;11(1):86. doi: 10.3390/vaccines11010086 (PMC9861981; doi:10.3390/vaccines11010086)
Supplement: Supplementary file 1 [file vaccines-11-00086-s001.zip › vaccines-1998482-supplementary.pdf]

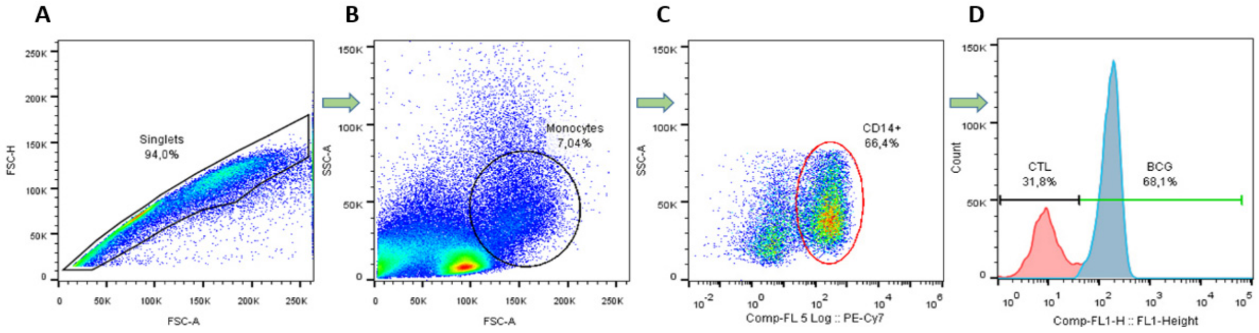

Figure S1. The gates strategy began with (A) a single cell inclusion at a time (Singlets), followed by (B) identification of the viable monocyte population on the basis of their light scattering properties by morphological characteristics using size (FSC) and complexity/granularity (SSC), subsequently, (C) a population of cells positive for the CD14 marker [84], and finally, (D) the TLR4, B7-1 DEC-1, TIM-3 or EP2-phenotypic marker expression, evaluated through comparative histograms between baseline, non-infected controls (CTL) and BCG-infected cells (BCG). In the dot plots, percentages of cells are indicated in each quadrant.

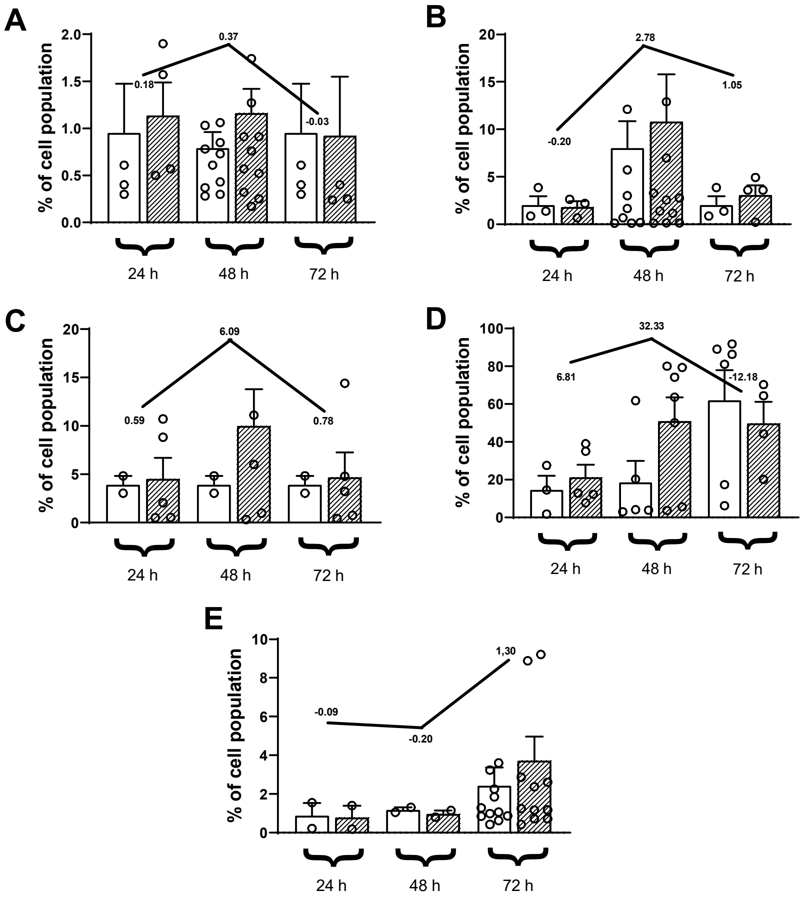

Figure S2. Time-point kinetic of (A) TLR4, (B) B7-1, (C) DEC-1, (D) TIM-3, and (E) EP2 expression levels on human mononuclears from healthy donor (HD; *n* = 5), as evaluated by flow cytometry. Bars (mean + SEM) stand for non-stimulated, baseline (opened) and BCG Moreau-infected cultures (hatched). Each line represents the delta (numbers) of in vitro BCG Moreau-infected *minus* non-stimulated, baseline cultures.

Table S1. The median and interquartile range phenotypic marker expression values (%) and marker intensity (MFI) induced in vitro on human mononuclears from adult healthy donors.

	%			MFI		
	**Baseline**	**BCG Moreau**	***p*-value**	**Baseline**	**BCG Moreau**	***p*-value**
**TLR4**	**0.67 (0.35-0.90)**	**1.09 (0.71-2.26)**	**0.014**	1.71 (1.60-2.51)	1.85 (1.10-2.45)	0.707
**B7-1**	**0.20 (0.12-0.36)**	**0.56 (0.18-1.92)**	**0.046**	1.73 (1.29-2.43)	1.51 (0.95-2.32)	0.459
**DEC-1**	**0.96 (0.22-1.75)**	**3.48 (1.44-20.77)**	**0.005**	3.54 (2.79-3.94)	3.63 (3.18-3.94)	0.554
**EP2**	**1.29 (0.85-2.25)**	**4.81 (1.45-28.73)**	**0.005**	1.48 (1.23-2.13)	1.50 (1.32-2.96)	0.416
**TIM-3**	31.90 (5.76-58.05)	32.30 (6.85-46.20)	0.869	3.56 (3.49-3.70)	3.64 (3.47-3.84)	0.604

Table S2. The median and interquartile range phenotypic marker expression values (%) and marker intensity (MFI) induced in vitro by BCG Moreau and baseline on human mononuclears from adult healthy donors (HD) and neonate (UCB) individuals.

	Baseline			BCG Moreau		
	**HD**	**UCB**	***p*-value**	**HD**	**UCB**	***p*-value**
%						
**TLR4**	0.67 (0.35-0.90)	1.10 (0.52-2.18)	0.096	1.09 (0.71-2.26)	1.46 (0.83-3.08)	0.331
**B7-1**	**0.20 (0.12-0.36)**	**1.04 (0.41-3.32)**	**<.001**	0.56 (0.18-1.92)	0.52 (0.35-2.58)	0.470
**DEC-1**	**0.96 (0.22-1.75)**	**3.54 (1.14-4.32)**	**0.011**	3.48 (1.44-20.77)	2.26 (1.02-3.67)	0.244
**EP2**	1.29 (0.85-2.25)	1.05 (0.55-2.30)	0.588	**4.81 (1.45-28.73)**	**0.89 (0.68-1.13)**	**<.001**
**MFI**						
**TLR4**	**1.71 (1.60-2.51)**	**1.32 (1.21-1.54)**	**0.016**	1.85 (1.10-2.45)	1.38 (1.19-1.49)	0.097
**B7-1**	**1.73 (1.29-2.43)**	**1.26 (0.85-1.34)**	**0.011**	1.51 (0.95-2.32)	1.30 (0.95-1.37)	0.291
**DEC-1**	2.79 (1.24-3.66)	2.87 (2.79-3.23)	0.730	3.33 (1.35-3.73)	2.95 (2.76-3.17)	0.383
**EP2**	1.48 (1.23-2.13)	1.37 (1.26-1.82)	0.466	1.50 (1.32-2.96)	1.42 (1.32-1.67)	0.220
